# Beta-Blocker Therapy After Myocardial Infarction: A Systematic Review and Meta-Analysis of Contemporary Randomized Controlled Trials

**DOI:** 10.3390/ijms27052363

**Published:** 2026-03-03

**Authors:** Stefania Angela Di Fusco, Andrea Matteucci, Alessandro Alonzo, Lorenzo Castello, Antonella Spinelli, Stefano Aquilani, Gaetano Marino, Silvio Fedele, Federico Nardi, Furio Colivicchi

**Affiliations:** 1 Clinical and Rehabilitation Cardiology Unit, San Filippo Neri Hospital, ASL Roma 1, 00135 Rome, Italy; 2 Cardiology Unit, Sandro Pertini Hospital, 00157 Rome, Italy; 3 Cardiology Unit, Santo Spirito Hospital, Casale Monferrato, 15033 Alessandria, Italy

**Keywords:** beta-blockers, meta-analysis, myocardial infarction, left ventricle ejection fraction, sex

## Abstract

The clinical benefit of beta-blocker treatment in patients with a previous myocardial infarction (MI) and without a reduced left ventricular ejection fraction (LVEF) is not established. This study aims at assessing the impact of beta-blocker treatment after an MI based on the type of MI at presentation, the LVEF, and the patient’s sex in the setting of contemporary management of MI. We searched the PubMed and Cochrane Library databases for randomized clinical trials published over last ten years that reported beta-blockers’ impact on prognosis in patients with LVEF > 40%. A meta-analysis was performed to assess the association between the beta-blocker treatment and outcomes in different patient subgroups based on the type at presentation (with ST segment elevation, STEMI, or without ST segment elevation, NSTEMI), LVEF, and sex. In the overall analysis, the association between beta-blocker non-use and the composite endpoint was not statistically significant under the random-effects model. In subgroup analyses, a higher risk with beta-blocker non-use was suggested in NSTEMI and in patients with mildly reduced LVEF in common-effect estimates (NSTEMI: RR 1.13, 95% CI 1.02–1.25; I^2^ 18%; mildly reduced LVEF: RR 1.24, 95% CI 1.03–1.49; I^2^ 0%), whereas corresponding random-effects estimates were not consistently significant. No clear association was observed in STEMI, preserved LVEF, or by sex. In sensitivity analyses excluding the ABYSS withdrawal trial, the overall association was attenuated and remained non-significant (random-effects RR 1.06, 95% CI 0.84–1.33; I^2^ 35%). Long-term beta-blocker therapy after myocardial infarction showed no clear overall benefit or harm across contemporary randomized trials. Possible signals of benefit in selected subgroups warrant confirmation in adequately powered studies with standardized endpoints.

## 1. Introduction

Despite a significant decline in myocardial infarction (MI) mortality in European Union countries over the last decade [1], ischemic heart disease remains the main cause of cardiovascular mortality [2]. The implementation of guideline-recommended treatments has led to less extensive ischemia-induced myocardial damage and better control of risk factors with a consequent significant reduction in the long-term incidence of cardiovascular events [3]. Due to the complexity of the modern approach to patients who have experienced an MI, the effective impact of pharmacotherapies introduced in clinical practice in the pre-reperfusion era, such as beta-blockers [4], that are supported by clinical studies performed when the interventional armamentarium was more limited, needs to be re-evaluated [5,6]. Among the long-term medical therapies traditionally used after MI, the role of beta-blockers in patients with left ventricular ejection fraction (LVEF) > 40% is one of the most debated issues. As regards the long-term clinical benefit of beta-blockers in this specific setting, observational studies and meta-analyses have given inconsistent results [7]. In recent years, randomized clinical trials (RCTs) have further evaluated the clinical impact of these drugs following an MI in the absence of a reduced LVEF. Scientific literature suggests a possible different impact of beta-blocker treatment based on the type of MI at presentation [8], the LVEF [9], and possible sex-specific effects [10]. In this systematic review and meta-analysis of recent RCTs, we assessed the impact of beta-blocker treatment after an MI based on the type of MI at presentation, the LVEF, and the patient’s sex.

## 2. Methods

### 2.1. Search Strategy

To identify RCTs that evaluated the impact of beta-blocker treatment on prognosis after an MI, we did a systematic review of the literature across PubMed/MEDLINE and the Cochrane Central Register of Controlled Trials (CENTRAL). The systematic review was performed in agreement with the Preferred Reporting Items for Systematic Reviews and Meta-Analysis (PRISMA) recommendations. The Medical Subject Headings (MeSH) terms entered were ‘beta blockers’ or “beta blocker” AND ‘after myocardial infarction’ or “post myocardial infarction”. The analysis was restricted to RCTs published in English over the last 5 years (up to September 2025). The clinical studies were included in the analysis if they met the following criteria: (1) randomized trials; (2) comparing beta-blocker treatment with no beta-blocker treatment; (3) included patients with a previous MI and a LVEF ≥ 40%; (4) patients enrolled in the study over the last ten years (2015–2025). We excluded studies that did not have major cardiovascular adverse events (MACE) as the primary endpoint.

All stages (literature search, study selection, data abstraction, risk-of-bias assessment, and statistical synthesis) were performed by two independent reviewers (SADF and AM). A third reviewer (F.C.) resolved discrepancies when needed.

For each eligible RCT, we collected trial design, sample size, mean age, sex distribution, follow-up duration, proportion of ST-segment elevation MI (STEMI) vs. non-STEMI (NSTEMI) patients, baseline LVEF, and treatment allocation. When available, subgroup data for STEMI versus NSTEMI, males versus females, and preserved versus mildly reduced LVEF were collected.

Risk of bias was assessed independently by two reviewers using the Cochrane Risk of Bias 2 tool, which evaluates the randomization process, deviations from intended interventions, missing outcome data, outcome measurement, and selective reporting. Disagreements were resolved by consensus (Supplementary Table S1).

### 2.2. Statistical Analysis

We performed all statistical analyses using the meta and metafor packages in R Studio (version 2025.09.0, Posit Software, Boston, MA, USA). Continuous variables were expressed as means and categorical variables as numbers and percentages. Dichotomous outcomes were analyzed as risk ratios (RRs) with corresponding 95% confidence intervals (CIs). For each trial, we extracted the number of composite primary endpoint events and denominators for both the primary endpoint of the trial and separately for all-cause death, myocardial infarction, stroke, and stratified groups (STEMI, or NSTEMI; LVEF between 40 and 50% or >50; and sex).

Meta-analyses were conducted with the Mantel–Haenszel method for fixed-effects models and the DerSimonian–Laird method for random-effects models. The Hartung–Knapp adjustment was applied for random-effects confidence. Heterogeneity was quantified using the τ^2^ statistic and I^2^, with I^2^ values of 25%, 50%, and 75% considered low, moderate, and high heterogeneity, respectively. Cochran’s Q-test was used to formally test for heterogeneity.

To evaluate robustness, we performed sensitivity analyses by sequentially omitting each trial (leave-one-out analysis), and we constructed Baujat plots to visualize the influence of individual studies on heterogeneity and effect size. Given the conceptual heterogeneity across the included randomized trials, we planned a sensitivity analysis excluding the ABYSS trial. While REDUCE-AMI, REBOOT, and BETAMI–DANBLOCK evaluate the strategies of initiating versus not initiating beta-blockers after acute myocardial infarction, ABYSS investigates late withdrawal versus continuation in stable patients already receiving beta-blocker therapy. Because these designs address related but distinct clinical questions (initial benefit vs. safety of withdrawal), excluding ABYSS allowed us to test the robustness of the pooled estimates when restricting the evidence to the initiation/no-initiation strategies. Publication bias and small-study effects were assessed by funnel plots, with asymmetry formally tested using regression-based methods (Egger and Harbord tests) and corrected estimates explored via the trim-and-fill method.

Subgroup analyses of betablocker-treatment impact were prespecified for STEMI versus NSTEMI patients, males versus females, and preserved versus mildly reduced ejection fraction. Separate pooled estimates were calculated within each subgroup, and a χ^2^ test for subgroup differences was performed to explore potential effect modification.

We conducted exploratory meta-regressions at the study level to examine whether mean age, proportion of male participants, and follow-up duration modified treatment effects. Meta-regressions were fitted using the metareg function in meta and, for confirmatory checks with small k, using metafor’s rma with REML estimation of τ^2^. Continuous moderators were modeled linearly; results are presented as regression coefficients on the log scale with 95% CIs and two-sided *p*-values. Because only four trials were available, all meta-regression findings are considered exploratory and hypothesis-generating. For these analyses, both univariable and bivariable models were tested, although we acknowledge the limited power given the small number of included trials (k = 4). Bubble plots were generated to visualize the association between covariates and log risk ratios.

## 3. Results

### 3.1. Search Results

**Figure 1:**
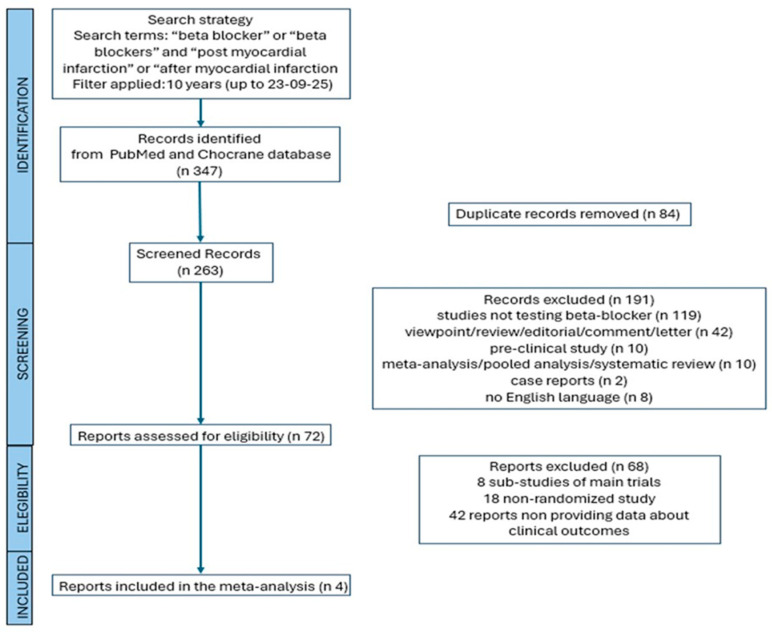
Preferred Reporting Items for Systematic Review and Meta-analysis flow-chart of studies included in the meta-analysis.

The search strategy is reported in Figure 1.

**Figure 2:**
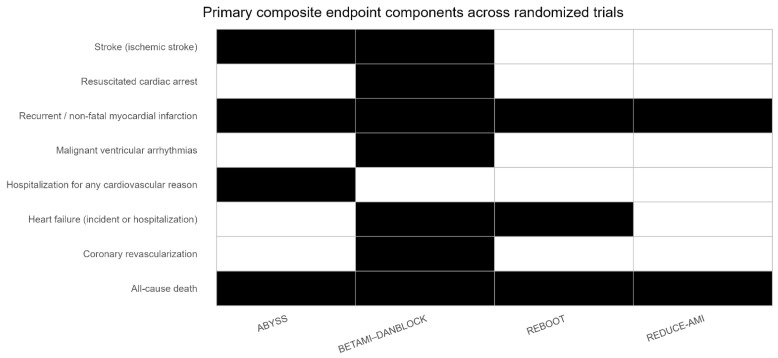
Comparative graphical representation of the components included in the primary composite endpoint of each randomized trial. Black cells indicate that the component is included in the endpoint definition for the corresponding study. Component labels were harmonized across trials to facilitate comparison (e.g., reinfarction/new non-fatal MI reported as “recurrent/non-fatal MI”; heart failure incidence or hospitalization grouped as “heart failure”). ABYSS evaluates a withdrawal strategy, whereas the other trials primarily address initiation/no-initiation strategies after myocardial infarction.

A total of four RCTs comprising 22,730 patients (11,380 non-treated with beta-blockers and 11,350 treated with beta-blockers) were included in the analysis. All trials included in this study enrolled patients over the last ten years (the eldest one, the Randomized Evaluation of Decreased Usage of Beta-Blockers after Acute Myocardial Infarction, REDUCE-AMI trial [11], started enrolment in September 2017); therefore, they evaluated the impact of beta-blocker treatment on top of the current optimal medical therapy. The characteristics of the studies included in the meta-analysis (REDUCE-AMI; the Treatment with Beta-Blockers after Myocardial Infarction without Reduced Ejection Fraction, REBOOT [12]; Assessment of Beta-Blocker Interruption 1 Year after an Uncomplicated Myocardial Infarction on Safety and Symptomatic Cardiac Events Requiring Hospitalization, AβYSS [13]; BETAMI-DANBLOCK [14] trial) and their main findings are reported in Table 1 and Figure 2.

### 3.2. Meta-Analysis Results

**Figure 3:**
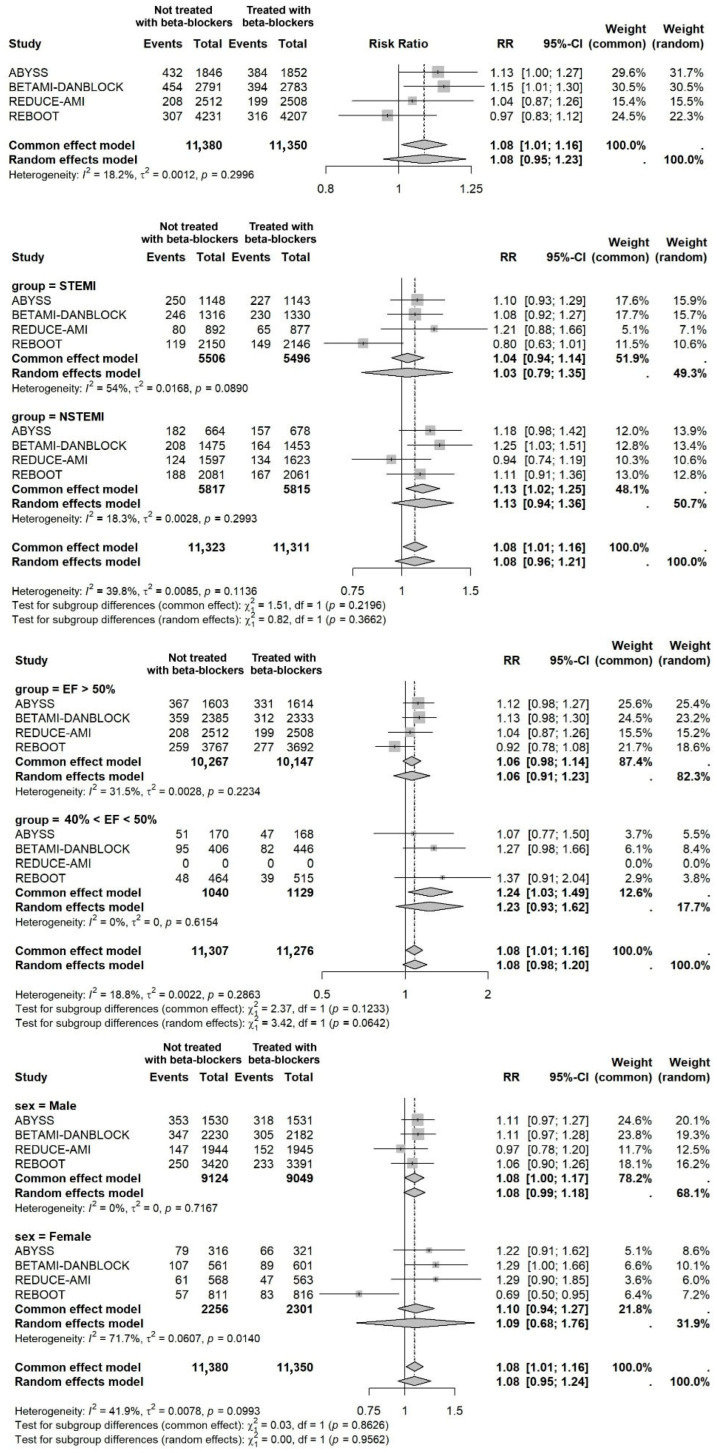
Forest plot of overall patient populations and subgroup analysis.

The overall analysis did not show a statistically significant association between beta-blocker non-use and the composite endpoint (random-effects RR 1.08, 95% CI 0.95–1.23; *p* = 0.14; fixed-effect RR 1.08, 95% CI 1.01–1.16; *p* = 0.027) (Figure 3). When assessed separately, no statistically significant associations were observed for the individual endpoints (all-cause death, myocardial infarction, and stroke). When stratified by type of MI, in STEMI patients, beta-blocker non-use showed no significant association with outcomes in either the random-effects model (RR 1.03, 95% CI 0.79–1.35) or in the common-effect model (RR 1.04, 95% CI 0.94–1.14; I^2^ = 54%). In NSTEMI patients, beta-blocker non-use did not show an increased risk in the random-effects (RR 1.13, 95% CI 0.94–1.36) and showed a minor correlation in the common-effect model (RR 1.13, 95% CI 1.02–1.25; I^2^ = 18%). A subgroup interaction test comparing STEMI and NSTEMI was not statistically significant (*p* = 0.37) (Figure 2). In analyses stratified by LVEF, beta-blocker treatment showed a neutral association in patients with preserved LVEF. Among patients with mildly reduced LVEF, the random-effects estimate was not statistically significant (RR 1.23, 95% CI 0.93–1.62) while the common-effect model reported a higher risk among those not treated with beta-blockers (RR 1.24, 95% CI 1.03–1.49; I^2^ = 0%) (Figure 2). When stratified by sex, no significant associations were observed in females (common-effect RR 1.10, 95% CI 0.94–1.27; I^2^ = 72%; random-effects RR 1.09, 95% CI 0.68–1.76), and the substantial heterogeneity indicates variability across studies (Figure 3).

Sensitivity analysis excluding ABYSS, restricting the evidence to initiation/no-initiation randomized trials (BETAMI–DANBLOCK, REDUCE-AMI, and REBOOT; k = 3), showed attenuation of the pooled association and no statistically significant effect on the composite endpoint (fixed-effect RR 1.07, 95% CI 0.98–1.16; random-effects RR 1.06, 95% CI 0.84–1.33; I^2^ = 35%). In subgroup analyses excluding ABYSS, estimates were overall neutral in patients with STEMI (RR 1.01, 95% CI 0.89–1.14), preserved LVEF > 50% (RR 1.03, 95% CI 0.94–1.13), and by sex (male RR 1.07, 95% CI 0.97–1.18; female RR 1.07, 95% CI 0.90–1.28) with random-effects model. In NSTEMI, the estimate became borderline and did not reach statistical significance (RR 1.12, 95% CI 0.99–1.26). In mildly reduced LVEF (<50%), results remained those observed in the comprehensive analysis. Overall, excluding the suspension strategy study did not affect the composite aggregate estimate without changing the results.

**Figure 4:**
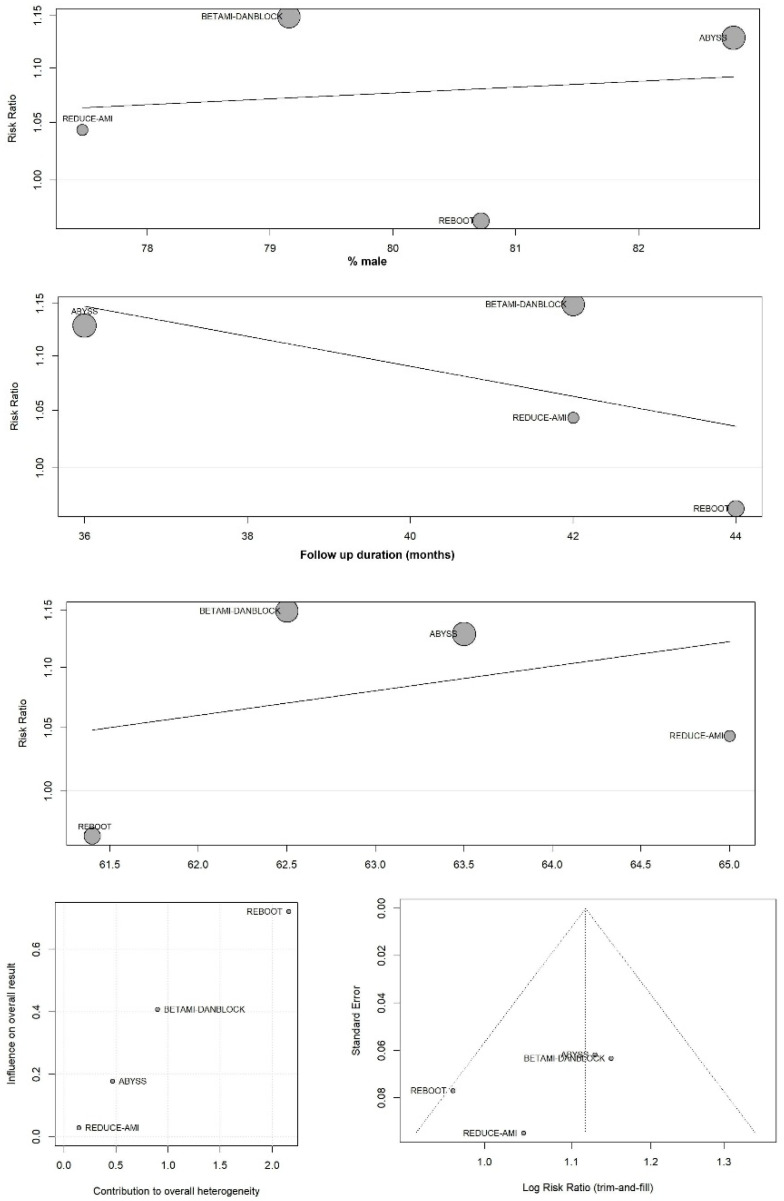
Meta-regression, Baujat plots, and funnel plot.

Exploratory meta-regressions using mean age, proportion of male participants, and study follow-up duration did not demonstrate significant effect modification; however, these analyses were underpowered given the small number of included trials (Figure 4). Leave-one-out analyses indicated that results were not driven by a single study, although statistical significance varied depending on the omitted trial. Baujat plots suggested that REBOOT contributed relatively more to heterogeneity but less to effect size, whereas REDUCE-AMI contributed proportionally more to the overall effect estimate. Funnel plots appeared visually symmetric, but the limited number of studies precluded formal assessment of small-study effects (Figure 4).

## 4. Discussion

The main findings of our meta-analysis on the impact of beta-blocker treatment on adverse outcome incidence after MI in patients with LVEF >40% include the following: the non-use of beta-blocker treatment as compared with beta-blocker treatment was found to be associated with a greater risk of the occurrence of adverse outcomes in NSTEMI patients while no significant clinical impact of beta-blocker treatment was found in patients with a previous STEMI; as regards the LVEF, a greater risk of incident major adverse events was found to be associated with the non-use of beta-blockers in patients with mildly reduced LVEF, but no significant impact of beta-blockers was found in patients with preserved LVEF, and neither was the beta-blocker treatment found to have a different impact in female and male patients.

An important aspect of interpreting these findings is the presence of conceptual heterogeneity across the randomized evidence. Specifically, REDUCE-AMI, REBOOT, and BETAMI–DANBLOCK primarily address the clinical decision of whether to initiate beta-blocker therapy after acute myocardial infarction, whereas ABYSS evaluates a different scenario, namely the safety of late withdrawal versus continuation in clinically stable patients already receiving beta-blockers. Although both topics relate to beta-blocker management after myocardial infarction, they reflect distinct clinical questions, initial benefit versus safety of discontinuation, and therefore should not be interpreted as fully interchangeable. Consistent with this distinction, when we restricted the analysis to trials evaluating initiation/no-initiation strategies by excluding ABYSS, the overall pooled effect for the composite endpoint was attenuated and did not show a clear statistically significant association. This suggests that part of the signal observed in the main pooled estimate may be influenced by combining trials addressing different management strategies, and highlights the need for careful contextual interpretation of pooled results.

An additional interpretative issue is the lack of standardization of primary endpoints across the randomized trials included in our quantitative synthesis. Although all studies address beta-blocker management after myocardial infarction, the composition of the primary composite outcome varies substantially, ranging from “hard” endpoints (all-cause death and recurrent myocardial infarction) to broader composites including heart failure events, coronary revascularization, stroke, malignant ventricular arrhythmias/resuscitated cardiac arrest, and hospitalization for cardiovascular reasons. Consequently, using the “primary outcome of each study” as the analytical unit introduces unavoidable endpoint heterogeneity, which may affect comparability and contribute to differences in effect estimates across studies.

Concerning the impact of long-term treatment with beta-blockers in STEMI patients without reduced LVEF, our meta-analysis results are consistent with the CAPITAL-RCT (Carvedilol Postintervention Long-term Administration in Large-scale Trial) study [15], which assessed the impact of beta-blockers (carvedilol) in STEMI patients (n 801) with LVEF ≥ 40%. As in our study, this RCT did not find a benefit of long-term beta-blocker treatment. The CAPITAL-RCT post hoc analysis, which separately analyzed data from patients with LVEF between 40 and 55% and those with LVEF ≥55%, also found a non-significant difference in the primary endpoint incidence between patients treated with beta-blockers and those not treated with beta-blockers. However, it is worth noting that a relevant limitation of the CAPITAL-RCT study is that, due to the difficulty in including patients, it was not adequately powered to detect a moderate impact of the treatment. A previous meta-analysis, which showed a lower all-cause mortality associated with beta-blocker treatment after STEMI [16], included no RCTs, and all the included studies were conducted between 1991 and 2011, when the standard pharmacological treatment post-MI did not include the same drugs as the contemporary treatment. A more recent meta-analysis of 16 observational studies that included MI patients (n 189,385) with a median LVEF of 53.7%, most of whom had a STEMI, showed no benefit of beta-blocker treatment on mortality [17].

In NSTEMI patients, observational studies have suggested the potential benefit of beta-blockers in selected subgroups, particularly at lower LVEF [18,19]. However, the signal observed under common-effect estimates was not consistently supported under random-effects, and no formal interaction supported a definitive difference between NSTEMI and STEMI. Therefore, any possible differential effect by MI type should be considered exploratory. In STEMI, historically associated with abrupt coronary occlusion and larger infarct size, the rationale for beta-blockers has been linked to the attenuation of sympathetic drive and reduction in myocardial oxygen demand through heart-rate and workload lowering [20]. However, in the contemporary era, early reperfusion strategies can limit infarct size and have been associated with the attenuation of adverse ventricular remodeling, potentially reducing the incremental prognostic impact of beta-blockade in many patients, particularly those who maintain preserved LVEF and therefore have a lower baseline risk profile [21]. Conversely, NSTEMI is frequently characterized by multivessel coronary artery disease and a higher likelihood of incomplete revascularization, which may sustain residual ischemic burden and “coronary vulnerability”; in this context, sympathetic modulation and heart-rate control may remain clinically relevant by reducing chronic myocardial demand in territories that are not fully revascularized [22,23].

Patients with mildly reduced LVEF represent a biologically plausible subgroup for benefit from β-blockade. In this intermediate range, residual neurohormonal activation and early adverse remodeling may persist despite contemporary reperfusion and background therapy, potentially translating into a higher vulnerability to heart failure progression and arrhythmic events compared with fully preserved LVEF. In support of this mechanistic plausibility, an individual patient–data meta-analysis focused on post-MI patients with mildly reduced LVEF reported a more consistent benefit signal of β-blockers in this subgroup [24]. More broadly, evidence from heart failure populations also indicates that β-blockers improve outcomes in patients with LVEF 40–49% (particularly in sinus rhythm), reinforcing the concept that modest systolic impairment may still be a “β-blocker-responsive” physiology [25]. The recent meta-analysis focusing on MI with mildly reduced LVEF, included individual patient data of two (REBOOT and BETAMI-DANBLOCK, 1835 patients) of four trials included in our study, in addition also included data from the CAPITAL-RCT (52 patients) and showed a lower risk of the primary MACE composite endpoint (all-cause death, MI, or heart failure) associated with beta-blocker treatment [24]. In our analyses, the direction of effect suggests a possible role of beta-blockers in mildly reducing LVEF, and this association persisted in sensitivity analyses restricted to initiation/no-initiation trials, whereas results were neutral in preserved LVEF. Nonetheless, given that the results under common-effect estimates were not supported under random-effects and considering the limited number of trials and differences in endpoint definitions, these findings warrant cautious interpretation.

Regarding sex-specific effects, our meta-analysis did not provide robust evidence of differential treatment effects between men and women. Point estimates were similar across sexes, but CIs were wide, and heterogeneity was substantial, indicating imprecision and inconsistency across studies. Therefore, sex-stratified findings should be considered uncertain. The REBOOT trial is the study that mainly impacts the heterogeneity of these results. Of note, the REBOOT trial included the largest cohort of women (n. 1162) in an RCT that tested beta-blockers after MI. Insights into the effect of beta-blockers in women with a previous MI without reduced LVEF are provided by the prespecified analysis of data from this trial [26]. This analysis showed a greater incidence of the primary outcome among women treated with beta-blockers than among those in the control arm (10.17% vs. 7.02%; hazard ratio 1.45; 95% CI: 1.03 to 2.03) and no meaningful difference in the primary outcome incidence among men, with a P for interaction suggestive of significant heterogeneity in the treatment impact across sex groups (*p* = 0.026). When stratified for LVEF, women treated with beta-blockers were found to have a higher incidence of the primary outcome (P for interaction by sex group 0.030) if they had a preserved LVEF. No treatment impact on the primary endpoint was found among women and men with mildly reduced LVEF (P for interaction 0.97).

### Study Limitations

The present study has several limitations. First, due to the aim of testing the impact of beta-blocker treatment on top of the current standard of care, we selected only RCTs that were conducted over the last decade. The number of studies included in this meta-analysis is limited, random-effects estimates were prioritized, and results should be interpreted cautiously. Second, clinical heterogeneity exists with respect to time from MI to randomization (from <7 days to >6 months), populations, and strategies tested (initiation/no-initiation vs. withdrawal). Third, primary composite endpoints were not fully uniform across trials. In the main text, we therefore added a graphical comparison of endpoint components to enhance transparency and support the correct interpretation of pooled estimates.

## 5. Conclusions

Our meta-analysis does not demonstrate a clear overall benefit or harm of long-term beta-blocker therapy after myocardial infarction in patients with LVEF >40%, in the context of clinical and endpoint heterogeneity across trials. Signals suggesting a possible association between beta-blocker non-use and a higher risk of adverse outcomes were observed in selected subgroups, particularly among patients with mildly reduced LVEF and, less consistently, in NSTEMI; however, these findings should be interpreted cautiously. Overall, the role of long-term beta-blockers after myocardial infarction in patients without reduced LVEF remains uncertain, and additional adequately powered trials with standardized endpoints are warranted to better define which patient profiles, if any, derive the greatest benefit.

## Figures and Tables

**Table 1:** Main characteristics and results of studies included in the meta-analysis [11,12,13,14].

Trial, Year * (Enrolment Period)	REDUCE-AMI, 2024 (2017–2023)	ABYSS, 2024 (2018–2022)	REBOOT, 2025 (2018–2024)	BETAMI–DANBLOCK, 2025 (2018–2024)
Population characteristics (n. patients randomized; proportion of female, %; proportion of STEMI, %; proportion of mildly reduced LVEF, %)	Recent MI (<7 days) having undergone early coronary angiography, LVEF≥ 50% (n. 5020; females, 22.5%; STEMI, 35.2%; mildly reduced LVEF, 0)	MI ≥ 6 months prior to randomization, LVEF ≥ 40%, without HF or arrhythmia or angina/ischemia requiring beta blockers (n 3698; females 17.2%; STEMI, 62.0%; mildly reduced LVEF, 9.1%)	MI managed with coronary angiography and LVEF > 40% before discharge, with no history of HF, contraindication to beta-blocker therapy, nor indication to betablockers different from the MI (8438 **; females 19.3%; STEMI, 50.9%; mildly reduced LVEF, 11.6%)	Patients with a MI within 7 ^§^ or 14 ^§§^ from presentation and LVEF > or ≥40% (5574 **; females 21%; STEMI, 47.5%; mildly reduced LVEF, 15.3%)
Study design	Registry-based, multicenter; 1:1 randomization; open-label	National (France), multicenter, 1:1 randomization, open-label, non-inferiority	Prospective, multicentric (Spain and Italy), 1:1 randomization, open-label, with blinded endpoint evaluation.	Prospective, multicenter (Denmark ^§^ and Norway ^§§)^, randomized, open-label, with blinded endpoint evaluation.
Treatment tested (n. patients in each treatment arm); median follow-up	Metoprolol (first choice) or bisoprolol (2508) versus no-beta blockers (2512)	Beta-blocker discontinuation (1846) versus continuation (1852)	Beta-blocker (4207) versus no beta-blocker (4231)	Beta-blocker (2783) versus no beta-blocker (4791)
Primary endpoint components	All-cause death, MI	All-cause death, MI, stroke, or hospitalization for cardiovascular reasons	All-cause death, MI, hospitalization for heart failure	All-cause death or MACE (MI, unplanned coronary revascularization, ischemic stroke, heart failure, or malignant ventricular arrhythmias).
Follow-up (median)	3.5 years	3 years	3.7 years	3.5 years
Main results	Beta-blocker treatment did not reduce primary endpoint incidence	Beta-blocker treatment discontinuation was not found to be non-inferior to beta-blocker continuation	Beta-blocker treatment did not impact the incidence of the primary endpoint	Beta-blocker treatment reduced the incidence of the primary endpoint

* Year of publication of the main study. ** Patients included in the main analysis ^§^ BETAMI. ^§§^ DANBLOCK. LVEF indicates left ventricle ejection fraction; MACE, major adverse cardiovascular event; MI, myocardial infarction; STEMI, myocardial infarction with ST-segment elevation.

## Data Availability

No new data were created or analyzed in this study. Data sharing is not applicable to this article.

## Supplementary Materials

The following supporting information can be downloaded at: https://www.mdpi.com/article/10.3390/ijms27052363/s1. Reference [27] is cited in the Supplementary Materials.

## Abbreviations

CI	confidence interval
LVEF	left ventricle ejection fraction
MI	myocardial infarction
mr-LVEF	mildly reduced left ventricle ejection fraction
NSTEMI	myocardial infarction without ST-segment elevation
p-LVEF	preserved left ventricle ejection fraction
STEMI	myocardial infarction without ST-segment elevation
RR	risk ratio
